# Lifetime imaging of GFP at CoxVIIIa reports respiratory supercomplex assembly *in live cells*

**DOI:** 10.1038/srep46055

**Published:** 2017-04-06

**Authors:** Bettina Rieger, Daria N. Shalaeva, Anna-Carina Söhnel, Wladislaw Kohl, Patrick Duwe, Armen Y. Mulkidjanian, Karin B. Busch

**Affiliations:** 1 Institute of Molecular Cell Biology, School of Biology, University of Münster, D-48149 Münster, Germany; 2 Mitochondrial Dynamics Group, School of Biology, University of Osnabrueck, D-49076 Osnabrueck, Germany; 3 School of Physics, University of Osnabrueck, D-49069 Osnabrueck, Germany; 4 School of Bioengineering and Bioinformatics, M.V. Lomonosov Moscow State University, 119992, Moscow, Russia; 5 A.N. Belozersky Institute of Physico-Chemical Biology, M.V. Lomonosov Moscow State University, 119992, Moscow, Russia

## Abstract

The assembly of respiratory complexes into macromolecular supercomplexes is currently a hot topic, especially in the context of newly available structural details. However, most work to date has been done with purified detergent-solubilized material and *in situ* confirmation is absent. We here set out to enable the recording of respiratory supercomplex formation in living cells. Fluorescent sensor proteins were placed at specific positions at cytochrome *c* oxidase suspected to either be at the surface of a CI_1_CIII_2_CIV_1_ supercomplex or buried within this supercomplex. In contrast to other loci, sensors at subunits CoxVIIIa and CoxVIIc reported a dense protein environment, as detected by significantly shortened fluorescence lifetimes. According to 3D modelling CoxVIIIa and CoxVIIc are buried in the CI_1_CIII_2_CIV_1_ supercomplex. Suppression of supercomplex scaffold proteins HIGD2A and CoxVIIa2l was accompanied by an increase in the lifetime of the CoxVIIIa-sensor in line with release of CIV from supercomplexes. Strikingly, our data provide strong evidence for defined stable supercomplex configuration *in situ*.

Under oxygenic conditions, mitochondria are pivotal for cellular ATP supply in oxidative phosphorylation (OXPHOS)^1^. The oxidative part mainly consists of three integral membrane protein complexes that are also proton pumps: Complex I (NADH dehydrogenase; CI), complex III (cytochrome *bc*_1_ complex; ubiquinol:cytochrome *c* reductase; CIII), and complex IV (cytochrome *c* oxidase; CIV). Assembly of these complexes into supercomplexes or respirasomes was already hypothesized in the 60′s of the last century based on electron microscopic images^2^ and later supported by biochemical analysis^3^,^4^. Structures were provided for CI_1_CIII_2_^5^ and CI_1_CIII_2_CIV_1_ assemblies^6^,^7^,^8^,^9^,^10^,^11^,^12^, supporting isolation-persistent supercomplexes assemblies. The assembly is supported by scaffold proteins, such as HIGD2A from the hypoxia inducible gene 1 (HIG1) family member and CoxVIIa2l (Cox7RP; SCAFI)^13^,^14^,^15^. However, several observations argue against stable supercomplex formation. First, the plasticity model demands flexible association – dissociation^16^, and second, single molecule and FRAP studies have shown that OXPHOS complexes are in principle mobile^17^,^18^. Furthermore, the functional relevance of supercomplex formation is still under debate^19^,^20^. Thus, a non-invasive live cell compatible technique that enables monitoring of dynamic supercomplex assembly *in situ* under live cell conditions, in addition to biochemical and genetic analysis methods, would be desirable. Here, we have implemented fluorescence lifetime imaging microscopy (FLIM) as such a method. FLIM is a fluorescence based technique takes advantage of the fact that the fluorescence lifetime τ of a fluorophore (defined as the average time that a molecule remains in the excited state after absorption of light prior to returning to the ground state) depends on its molecular environment. Fluorescence lifetime determination is therefore a feasible means of monitoring the local environment of proteins, such as the association of proteins into complexes and the re-location of proteins between different cellular micro-compartments^21^,^22^,^23^,^24^,^25^.

Here, we set out to prove that FLIM might also be a suitable technique to monitor respiratory supercomplex assembly in live cells.

## Results

To test this hypothesis, we designed several lifetime probes by attaching fluorescent proteins to specific respiratory subunits which are embedded in a supercomplex based on structural information. We anticipate that positioning a fluorescence probe in a crowded environment, dense with proteins, should correlate with a shortened lifetime due to multiple dipole-dipole interactions. As a FLIM sensor probe, we used the fluorescent protein superecliptic pHluorin (sEcGFP), a pH sensitive monomeric EGFP variant (F64L, S65T, S147D, N149Q, V163A, S175G, S202F, Q204T, A206T) referred to as sEcGFP^26^. The fluorescence lifetime τ was determined in the time-domain by time-correlated single photon counting (TCSPC) fluorescence lifetime imaging microscopy (FLIM). Soluble purified sEcGFP in PBS had a lifetime of τ = 2.23 ns analogous to that reported for GFP^27^. In aqueous solutions with increasing glycerol concentrations, which mimic increasing molecular crowding, the lifetime of sEcGFP decreased as expected (Supplementary Fig. S1)^28^.

### Probes at CoxVIIIa and CoxVIIc report supercomplex formation

**Figure 1:**
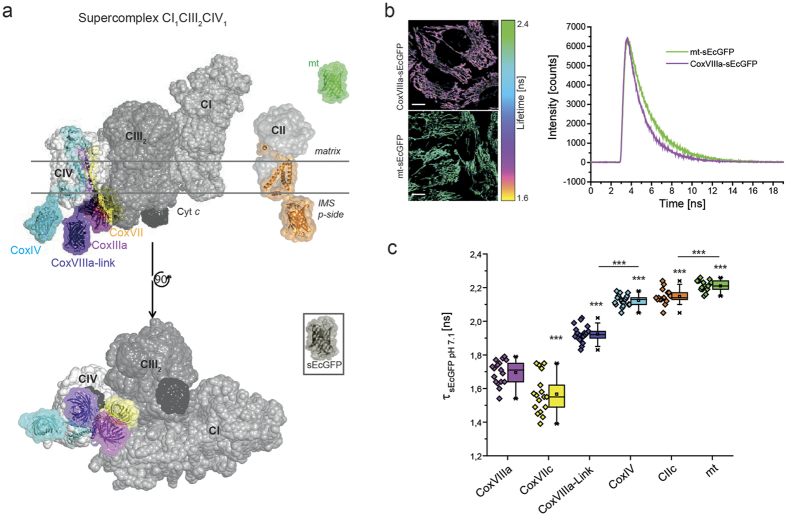
Lifetime variations of sEcGFP attached to OXPHOS subunits CoxVIIIa and CoxVIIc report specific molecular crowding at the contact site of CI_1_CIII_2_CIV_1_. (**a**) 3D maps of CII and the CI_1_CIII_2_CIV_1_ supercomplex^7^ showing the side view (top) and the view of the supercomplex from the *p*-side (complex I in grey, complex III in darker grey and complex IV in light grey, cytochrome *c* in black). sEcGFP was fused to different subunits of respiratory complexes: CoxVIIc (yellow), CoxVIIIa (pink), CoxVIIIa-Link (violet), CoxIV (cyan), CIIC/SDHC (orange) and to a short matrix targeting sequence mt (green). Selected fitting models are shown. (**b**) Intensity lifetime image and time correlated single photon counting (TCSCP) histograms of mt-sEcGFP and CoxVIIIa-sEcGFP showing clear differences in lifetime revealed from the decay of fluorescence lifetime. Scale bars: 10 μm. (**c**) Lifetime constants τ for sEcGFP at different subunits of cytochrome *c* oxidase (CoxVIIIa - pink, CoxVIIc - yellow, and CoxIV - cyan), at subunit C of SDH (orange) and of matrix-targeted soluble sEcGFP (green). Each data point in the box-and-whisker plot represents the average fluorescence lifetime of a cell ( = one mitochondrial network). The error bars denote s.d., the boxes represent the 25^th^ to 75^th^ percentiles. The vertical lines in the boxes represent the median values, whereas the square symbols in the boxes denote the respective mean values. The minimum and maximum values are denoted by x. All measurements were performed at pH 7.1 to exclude pH effects^28^. One data point per cell, error bars represent s.d. of ∼18 cells (n = 3 replicates). ***P < 0.001 (by one-way ANOVA).

We then tested the appropriateness of FLIM to detect supercomplex formation, which is a specific form of molecular crowding, *in situ*. Therefore, sEcGFP was fused to the C-terminus of subunit CoxVIIIa of cytochrome *c* oxidase in the inner mitochondrial membrane (Fig. 1a, pink structure)^29^. According to previous and recent structures, CoxVIIIa is buried at the interface between complexes I, III and IV. As a control, a matrix-targeted mt-sEcGFP was generated (Fig. 1a, green structure). Lifetime intensity images from cells expressing either of the constructs showed a clear difference (Fig. 1b, left panel). The corresponding time constant τ of the sEcGFP fluorescence lifetime decay was determined by fitting the respective TCSPC diagram (Fig. 1b, right panel). For soluble mt-sEcGFP, a mono-exponential fit can be used^30^, while for the membrane bound form, a bi-exponential fit was more appropriate (Supplementary Fig. S2). The averaged lifetime for CoxVIIIa-sEcGFP τ_amp_ = 1.69 ns was significantly lower than for mt-sEcGFP with τ_amp_ = 2.21 ns (δ_amp_ = 0.52 ns). To test the possible influence of pH on the result (basic matrix pH and acidic intermembrane space pH), the lifetime of both constructs was recorded in cells at different pH values in the range of pH 6.1 to pH 8.5 as described before^29^. The lifetime differences due to altered pH were small in the physiological range (pH 7–8), and thus were not responsible for the lifetime differences between mt-sEcGFP and CoxVIIIa-sEcGFP (Supplementary Fig. S3).

The low fluorescence lifetime τ_amp_ of CoxVIIIa-sEcGFP was therefore relevant and independent of pH. When fitted into a 3D model of supercomplex, the CoxVIIIa-sEcGFP sensor is buried between CIV and CIII (Fig. 1a, pink barrel). As a consequence, multiple dipole-dipole interactions with adjacent proteins from complex I and III are possible that could account for the lifetime reduction of the excited state for sEcGFP at this specific CoxVIIIa position.

We next tested CoxVIIc as a further candidate to probe supercomplex formation since it is probably also exposed to a high protein density (Fig. 1a, yellow)^10^. sEcGFP at CoxIV was used as a control as it is located at the surface of the supercomplex where protein density probably is lower (Fig. 1a, light blue; Supplementary Fig. S4). To exclude interference with metabolic activity, pH, or redox potential, cells transfected with the respective sEcGFP-subunits were permeabilized, the OXPHOS activity was inhibited and the pH set to pH 7.1 during FLIM measurements. Biexponential fits of TSCPC decays were more appropriate than mono-exponential fits and thus further used for determination of lifetimes (Supplementary Fig. S2). Strikingly, the lifetime of the CoxVIIc sensor was very short (τ = 1.57 ± 0.12 ns, s.d.), while the lifetime of the sensor at CoxIV was significantly longer (τ = 2.12 ± 0.03 ns, s.d.) than that for CoxVIIIa and CoxVIIc. The calculated distance of sEcGFP to the last amino acid of the respective transmembrane domain is 1–2 Å in CoxVIIc-sEcGFP and 5 Å in CoxVIIIa-sEcGFP, which implies positions buried deep in the supercomplex. To elevate the sensor out of the supercomplex, we next inserted a linker composed of the C-terminal sequence of CoxVIIb between CoxVIIIa and sEcGFP (Table 1). The introduction of the linker resulted in a significantly longer lifetime τ = 1.92 ns ( ± 0.05 ns, s.d.) of the fusion protein CoxVIIIa-link-sEcGFP, which let us conclude that this sensor attached via a linker could escape from the crowded protein environment of the supercomplex (Fig. 1a, CoxVIIIa-link, violet).

**Figure 2:**
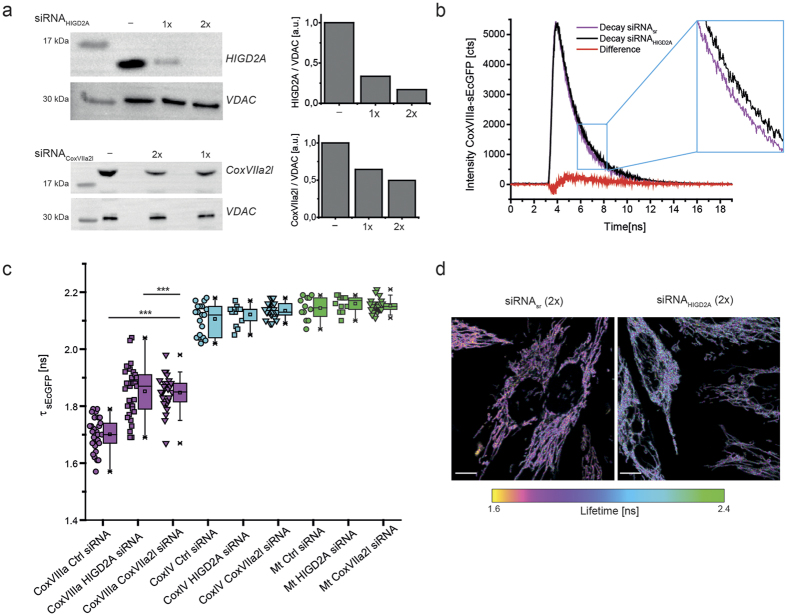
The lifetime of CoxVIIIa-sEcGFP increases in cells with decreased levels of scaffold proteins. (**a**) Silencing of HIGD2A and CoxVIIa2l in stable CoxVIIIa-sEcGFP cells as shown by immuno-staining. Scrambled siRNA was used as a control, loading control was VDAC (middle panel). Right panel: ratio between HIGD2A respectively CoxVIIa2l and VDAC levels. (**b**) TCSPC diagram showing the change in fluorescence decay with decreased scaffold proteins. (**c**) Average fluorescence lifetimes τ_amp_ of stable HeLa cell lines expressing CoxVIIIa-sEcGFP, CoxIV-sEcGFP and mt-sEcGFP, respectively with downregulated HIGD2A or CoxVIIa2l. One data point per cell, error bars represent s.d. of ∼24 cells (n = 3 biological replicates). (**d**) Fluorescence intensity/lifetime images of CoxVIIIa-sEcGFP in cells with decreased scaffold protein HIGD2A or All-Stars non-targeting siRNA. Scale bars: 10 μm. Significance: ***P < 0.001 compared to CoxVIIIa-sEcGFP (ANOVA one-way).

To verify that the decreased lifetime of sEcGFP at CoxVIIIa was an effect of defined supercomplex assembly but not caused by random molecular crowding or by the short distance of the probe to the membrane surface, we determined the lifetime of sEcGFP attached to subunit C of complex II. OXPHOS complex CII is a monomeric protein complex^16^ found in cristae as CI, CIII and CIV^31^. sEcGFP was fused to the C-terminus of the C-subunit of CII (SDHC) with a 7 amino acid residues linker that should result in a short distance between the probe and the membrane surface (Table 1). The resulting fluorescence lifetime τ = 2.15 ns ( ± 0.05 ns, s.d.) for CIIC-sEcGFP was similar to the lifetime of CoxIV- sEcGFP (τ = 2.12 ns ± 0.03 ns, s.d.) at the surface of CIV. It was about 0.47 ns higher than that for CoxVIIc-sEcGFP with the same linker length between the membrane domain and the sEcGFP (Table 1). Overall, the fluorescence lifetimes of CoxVIIc-sEcGFP and CoxVIIIa-sEcGFP were the shortest of all lifetimes measured by a significant margin, and this is consistent with a position in the center of a supercomplex (Fig. 2c). Similar results were obtained with mCitrine as sensor – except that the τ values were shifted to longer τ(Δ~0.59 ns) due to the generally longer lifetime of mCitrine (Supplementary Fig. S5)^32^.

Because mitochondria of cells expressing CoxVIIc-sEcGFP appeared stressed with few mitochondria in the cell periphery (data not shown) and slightly decreased basal and ATP-linked respiration (Supplementary Fig. S6), we decided to use CoxVIIIa-sEcGFP for further studies. In stable CoxVIIIa-sEcGFP cells no alterations of mitochondrial morphology, distribution or respiration rates were observed (Figure 1b, Supplementary Fig. S6). Successful assembly of a CoxVIIIa-GFP fusion construct into complex IV was shown earlier^33^.

### Influence of supercomplex scaffold factors

We next decreased levels of supercomplex scaffold factors HIGD2A and CoxVIIa2l by siRNA treatment. Twofold siRNA treatment resulted in 80% decreased HIGD2A levels, while CoxVIIa2l levels were decreased to 50% (Fig. 2a). The TCSPC decays of CoxVIIIa-sEcGFP in cells with decreased scaffold factor HIGD2A showed a significant shift towards longer lifetimes (Fig. 2b). The HIG2DA suppression obviously did not influence the cellular or mitochondrial morphology (Fig. 2d, lower panel).

**Figure 3:**
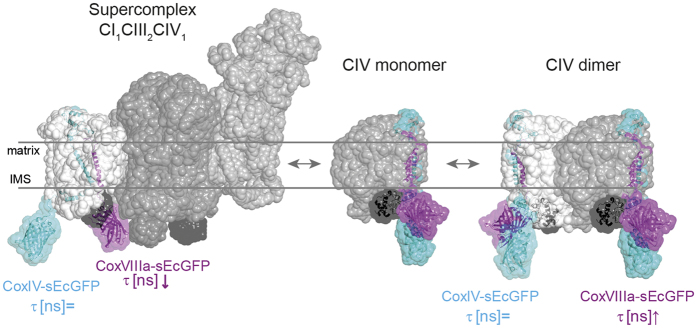
Positioning of CoxIV-sEcGFP and CoxVIIIa-sEcGFP in a supercomplex and monomeric and dimeric CIV. Models illustrating conformations expected for CoxVIIIa-sEcGFP in a supercomplex and in monomeric and homo-dimeric CIV_(2)_ with respective environments: CoxVIIIa-sEcGFP senses a dense molecular environment in a supercomplex and an aqueous environment in free CIV_(2)_ and CIV, while CoxIV-sEcGFP is always exposed to an aqueous environment. Supercomplex sensor CoxVIIIa-sEcGFP in pink framed with a dotted line, control CoxIV-sEcGFP in cyan, cytochrome *c* framed with disrupted lines.

Overall, the suppression of the supercomplex assembly factors HIGD2A and CoxVIIa2l increased the mean lifetime of the CoxVIIIa probe but not of the CoxIV- or mt-sEcGFP probe (Fig. 2c). Moreover, according to the residuals (Supplementary Fig. S2) mono-exponential fits for CoxVIIIa-sEcGFP with suppressed HIGD2A and CoxVIIa2l became appropriate in most cases. This is what we had expected as an outcome of supercomplex disassembly and CoxVIIIa-sEcGFP release. While CoxVIIIa-sEcGFP in a supercomplex will be subject to a dense protein environment, CoxVIIIa-sEcGFP in monomeric cytochrome *c* oxidase or in cytochrome *c* oxidase homo-dimers will rather be exposed to bulk water, resulting in less dipole-dipole interactions and thus an increase in the corresponding fluorescence lifetime of CoxVIIIa-sEcGFP (Fig. 3). CoxVIIIa-sEcGFP is localized at the outward surface, since subunits VIa, VIb and VIIb are the subunits that form the major protein-protein contacts between the two monomeric units within the dimeric complex (Supplementary Fig. S7)^34^,^35^,^36^. We thus interpret the prolonged fluorescence lifetime in HIGD2A down-regulated cells as a shift towards higher amounts of free CIV and less CoxVIIIa-sEcGFP buried within a supercomplex. However, the lifetime of CoxVIIIa-link-sEcGFP was not changed in cells with downregulated HIGD2A (Supplementary Fig. S8). In general, the increase of τ_amp_ was not accompanied by an increase in fluorescence intensity (Supplementary Fig. S9) which is not surprising since fluorescence intensity is not necessarily a parameter that responds to the nano-environment^28^. There is also no possibility of using fluorescence intensity read out for quantification, since expression levels of the different fluorescent fusion-proteins were not comparable (Supplementary Fig. S9).

In summary, our live cell FLIM approach with probes at different Cox subunits clearly demonstrates the exceptional position of subunit CoxVIIIa (and CoxVIIc) and is consistent with localization of CIV inside a supercomplex but not by CIV monomers or dimers (Fig. 3).

### FRET between CIII and CIV confirms supercomplex formation

**Figure 4:**
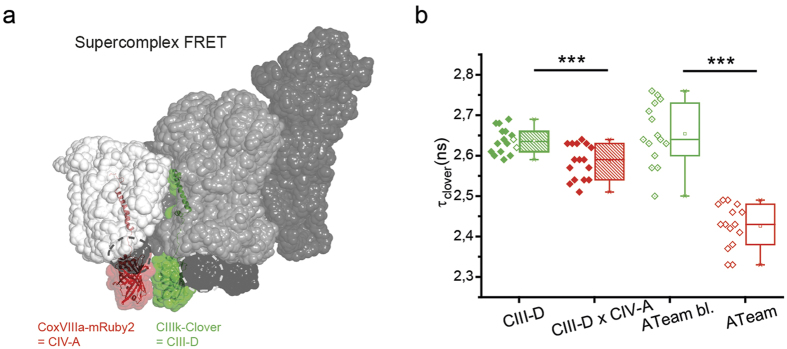
FRET measurements showing close proximity of CIV subunit CoxVIIIa and CIII-subunit k in a supercomplex. (**a**) Positions of CoxVIIIa-mRuby (acceptor, CIV-A) and CIIIk-Clover (donor, CIII-D) within a CICIII_2_CIV supercomplex. Only one subunit k of one CIII is labeled. (**b**) Fluorescence lifetime of Clover as donor in the presence and absence of acceptor mRuby. Pair 1: CoxVIIIa-mRuby as acceptor and CIIIk-clover as donor. Pair 2: modified ATeam^37^ composed of clover as donor and mRuby as acceptor was used as FRET control. Significance: ***P < 0.001 compared to CIIIk-Clover (ANOVA one-way).

We next tested possible colocalization of CIII and CIV in a supercomplex by Förster Resonance Energy Transfer (FRET) using CIII subunit k fused to Clover as donor and CoxVIIIa-mRuby2 as acceptor. The C-terminus of CIII k is localized at the p-side according to the supercomplex models and the distance between the fused Clover and CoxVIIIa-mRuby2 should be short enough to allow FRET (Fig. 4a). Cells were transiently transfected with donor only (CIIIk-Clover, CIII-D) or both constructs (CIII-D × CIV-A) and fluorescence lifetime images were recorded. As a control, ATeam^37^ was used that was constructed with Clover as donor and mRuby2 as acceptor within the same ATeam molecule. The lifetime of Clover was obtained from bi-exponential fits from the TCSPC decays. In the presence of the acceptor mRuby2, the lifetime of the donor significantly decreased. This indicates proximity of CIII and CIV in a supercomplex. The decrease in fluorescence lifetime was stronger in an ATeam control construct, since here the expression and proximity of both fluorescent proteins was guaranteed (Fig. 4b, Supplementary Fig. S10). The FRET results thus are in line with the preceding FLIM data with one sensor only at CoxVIIIa and suggest the existence of a CICIII_2_CIV supercomplex *in situ*.

## Discussion

The architecture of respiratory supercomplexes has been revealed and refined by several groups^6^,^7^,^8^,^9^,^10^,^11^,^12^. Purification and re-constitution involve some critical steps^16^ and freeze the supercomplex in a certain state^38^. Thus, dynamic supercomplex assembly is difficult to determine from the analysis of purified supercomplexes. Notably, approximately 80% of total CI, and 66% of total CIII_2_ was found to be supercomplexed, but 85% of total CIV – according to BN-PAGE data – was found in the free form in bovine heart mitochondria^39^. This probably makes CIV_1_ the more dynamically regulated complex. For that reason, we focused on CIV_1_ for *in situ* studies. Moreover, it was reported that interactions between CIV and CI/CIII were weaker than interactions between CI and CIII^9^. By FLIM using fluorescent sensor proteins at specific positions we provide a tool to study supercomplex assembly in live cells and monitor supercomplex plasticity. We focused on single probes since FRET is much more difficult and not straight forward for several reasons: (i) The successful labeling of two subunits within one supercomplex for successful FRET studies is more challenging than working with a single sensor, (ii) The recording time after transfection is critical, since little is known about the turnover and assembly of SU subunits into complexes and supercomplexes, (iii) Attachment of fluorescent proteins to two subunits might interfere with supercomplex assembly, (iv) CIII_2_ was always found as a dimeric complex and this might complicate the analysis. However, we were able to obtain positive FRET between labeled subunits CoxVIIIa of CIV and subunit k of CIII indicating proximity within the Förster radius. FRET was used before to show F_1_F_O_ ATP synthase interaction^40^. Instead, we decided to go for a simpler assay: the lifetime of a single fluorescent probe already reports on the local molecular environment and becomes shorter in protein dense environments. We genetically sited fluorescent proteins to different subunits of cytochrome *c* oxidase (CIV) for FLIM analysis. Lifetime measurements with fluorescent proteins are feasible since interactions with proximal proteins are translated into the interior of the fluorescent protein barrel and decrease the lifetime^41^. CIV is part of several supercomplexes described to date with specific configurations^3^,^4^. Assembly of complexes into a supercomplex explains the finding that individual complex stability depends on the interaction with other complexes^42^,^43^,^44^ better than random collision in a crowded membrane. Also, the isolation of mammalian supercomplex CI_1_CIII_2_CIV_1_ indicates a rather stable supercomplex association with *K*_D_ values in the ms to s time range^39^. The short sEcGFP fluorescence lifetimes that we measured at subunits CoxVIIIa and CoxVIIc of cytochrome *c* oxidase fit exactly into the models of the CI_1_CIII_2_CIV_1_ supercomplexes, indicating a dense protein environment at the interface between CIV, CI and CIII. From our results, we can rule out random collision as the general cause for the observed lifetime reduction, since the fluorescence lifetime of CIIC-sEcGFP in the same microcompartment was significantly higher than of CoxVIIIa/CoxVIIc-sEcGFP. Rather, the shortened lifetimes of the CoxVIIIa and CoxVIIc sensors is caused by higher protein density at their location due to a specific, defined protein environment in a supercomplex. This was supported by the finding that suppression of supercomplex scaffold factors specifically increased the lifetime of CoxVIIIa, the sensor embedded within the supercomplex, but not of sensors that were located only at the surface or a sensor attached with a long link to the CoxVIIIa subunit. This is in line with a shift to monomeric and dimeric forms of CIV^45^.

Our data show that FLIM with one sensor is a valuable alternative to the Förster Resonance Energy Transfer technique. Not only, FLIM is a tool to investigate supercomplex formation and dissociation non-invasively in a functional context but has the potential to be used to study other events involving complex formation such as G protein coupled signalling, or supercomplex formation in photosynthetic membranes.

## Material and Methods

### FLIM sensor construction

For eukaryotic expression of sEcGFP, the full-length protein-coding region of superecliptic pHluorin (F64L/S65T/S147D/N149Q/V163A/S175G/S202F/Q204T/A206T) (a gift from Prof. Jürgen Klingauf) was inserted by PCR amplification into a modified pSEMS-26 m vector from NEB Biosciences (formerly Covalys Biosciences). For primers see Rieger *et al*. 2014^29^. For determination of the fluorescence lifetime in the matrix bulk, the mitochondrial targeting sequence of the mitochondrial processing peptidase MPP, consisting of the N-terminal 60 amino acid residues (MPP60, referred to as mt), was included behind the CMV promoter and at the 3′end of sEcGFP in this vector resulting in mt-sEcGFP. This targeting sequence is likely removed from the fusion construct by MPP, leaving sEcGFP in the matrix. For measurements in the IMS, CoxVIIc-, CoxVIIIa-, CoxIV-, and CIIC-sEcGFP were assembled behind the CMV promoter and at the 3′end of sEcGFP in the sEcGFP-vector. The respective subunits of OXPHOS complexes were fused with their C termini to the N terminus of sEcGFP. To increase sensor distance to the membrane by C-terminal extension of CoxVIIIa, we used part of the C-terminal sequence of CoxVIIb (TCCCCTGTTG GCAGAGTTAC CCCAAAGGAA TGGAGGAATC AG) to insert a linker between CoxVIIIa and sEcGFP, resulting in CoxVIIIa-Link-sEcGFP. For FLIM measurements in the presence of an acceptor, CoxVIIIa-mRuby2 was generated as an acceptor, while subunit k of CIII was C-terminally fused to Clover as donor.

### Construction of pSems-Clover-ATeam-mRuby2

In CFP-ATeam-YFP (AT1.03; a gift from Hiromi Imamura)^37^ CFP was substituted by Clover and YFP was substituted by mRuby2. Therefore, primers for ATeam, Clover and mRuby2 were constructed: f-ATeam-BamHI GATTAGGGATCCATGAAAACTGTGAAA GTGAATATAAC, r-ATeam-XhoI GATTAGCTCGAGGTTTGCCTTCCCAGCCACGTCCAG, f-mRuby2-XhoI GAT TAGCTCGAGATGGTGTCTAAGGGCGAAGAGC, r-mRuby2-NotI GATTACGCGGCCGCCTTGTACAGCTCGTCC ATCC. ATeam and mRuby were amplified by PCR and inserted into a modified pSEMS-MCS vector from NEB Biosciences (formerly Covalys Biosciences). Therefore, the ATeam PCR construct was digested with BamHI and XhoI and the mRuby2 PCR construct with XhoI and NotI. Finally, Clover from pSems-FRB-Clover (cloning analogous to pSems-FRB-mCitrine^8^ was inserted in pSems-ATeam-mRuby2 after digestion with EcoRI and BamHI. PCR primers were purchased from Sigma Aldrich. Enzymes, restriction buffer and BSA were purchased from NEB Biosciences.

### Cell culture of HeLa cells

For generation of stable cell lines, transfected HeLa cells were selected for stable neomycine resistance by growth in the presence of 0.8 mg/ml G418 (Calbiochem 345810). Untransfected HeLa cells and the stable HeLa cell lines (CoxVIIc-, CoxVIIIa-, CoxVIIIa-Link-, CoxIV- and mt-sEcGFP) were cultured in Minimal Essential Medium with Earle’s salts (MEM, PAA Lab GmbH, E15–888) with 5.6 mM glucose, 2 mM stable glutamine and sodium bicarbonate, supplemented with 10% (v/v) fetal bovine serum (FBS) superior (Biochrom AG), 1% MEM nonessential amino acids (NEAA, Biochrom AG) and 1% 4−(2−hydroxyethyl)piperazine-1-ethanesulfonic acid (HEPES, PAA Lab GmbH) at 37 °C with 5% CO_2_. Stable HeLa cell lines were kept in 0.8 mg/ml G418 in addition. Cells were split 2–3 times a week using Trypsin/EDTA (Biochrom), supplemented with HEPES (PAA), sodium bicarbonate (PAA), penicillin/streptomycin (Biochrom) and PBS (Biochrom). For nanoenvironment determination by fluorescence lifetime measurements, sEcGFP cell lines were seeded onto glass coverslips in 3.5 cm cell culture dishes. Imaging was performed 2 days after seeding.

### siRNA mediated knockdown

HeLa cells were maintained in culture medium. Knockdowns were performed by treating cells with 5 nM total siRNA (Qiagen), using the Hiperfect transfection reagent, according to the manufacturer’s instructions for fast-forward transfection (Qiagen). The All-Stars non-targeting siRNA (1027295/SI03650318) was used as the control for siRNAs targeting HIGD2A (NM_ 138820, 1027419/SI04275957) and CoxVIIa2l (NM_004718, 1027419/SI00067445) – all with 3′ AlexaFluor647 modification. Cells were subjected to knockdown on day zero, again on day 1, and analysed on day 3.

### SDS-PAGE analysis

For SDS-PAGE, cell lysates from samples of confluent T-25 flasks (1 flask would be sufficient for 10–15 gels) were heated at 95 °C for 5 min and separated on 12% Tricine-SDS-PAGEs and transferred to PVDF membranes. Membranes were blocked prior to detection using 10% skimmed milk powder in TBS-T (200 mM Tris, 1.37 M NaCl, + 0.1% Tween20). BN-PAGE was performed using a Tricine-Tris buffer system. SuperSignal West Pico from ThermoScientific (34080) was used as chemiluminescent substrate to visualize proteins. Protein ladder was purchased from ThermoScientific (ThermoScientific PageRuler Plus Prestained Protein Ladder #26619). Semi-Dry-Blotting was performed using a Tris-Glycine buffer with 13 mM SDS. Antibodies were purchased from CellSignaling (VDAC, D73D12 #4661), antikoerper-online.de (HIG1 Hypoxia Inducible Domain Family, Member 2 A (HIGD2A) (AA15–44) (N-Term) antibody, ABIN658252), Abcam (CoxVIIa2l, ab170696) or Dianova (Peroxidase-conjugated AffiniPure Goat Anti-Rabbit IgG (H + L), 111-035-045).

### Time correlated single photon counting (TCSPC) fluorescence lifetime imaging microscopy (FLIM)

Time-resolved fluorescence measurements were recorded *in situ* at 37 °C and 5% CO_2_ using a confocal laser scanning microscope (FluoView FV1000, Olympus) equipped with a TCSPC extension module (PicoQuant GmbH). The excitation source was a pulsed LDH-D-C-485 laser (PicoQuant GmbH) operated at a repetition time rate of 40 MHz. The output pulses were coupled into an optical fiber. The output at the fiber end was reflected from a beam splitter onto the base of a multiwell plate (Ibidi^®^, 30 μL) or a glass cover slip via a 60 × oil-immersion objective (UPLSAPO oil, NA 1.35, N/0.17/FN26.5, Olympus) upgraded with an objective heater (BIOPTECHS). Emission was restricted to 525/50 nm with an emission filter and photons were detected with a single photon avalanche diode (SPAD). The acquisition was performed until at least 1000 photons in the brightest pixel were reached. For calibration, sEcGFP-GST was expressed in *E.coli* and purified via agarose beads. The purified sEcGFP was added to aqueous Dulbecco’s Phosphate Buffered Saline (PBS) solutions with increasing glycerol content (w/w%) at 37 °C. In parallel, the refractive index of the same glycerol/PBS mixtures was determined by an Abbe-refractometer heated to 37 °C. Data analysis was performed with SymphoTime^®^ software (32 bit) and mono- respectively bi-exponential fitting of the fluorescence decay curves (substracting the IRF) from ROIs (approx. 50% of the mitochondrial network of a cell). From bi-exponential fits, the average lifetime was calculated as mean weighted average lifetime τ_amp_ = ((τ1.A1) + (τ2.A2))/(A1 + A2), with τ1 and τ2 as different lifetimes and A1 and A2 as their amplitudes. For measurement of sEcGFP proteins in HeLa cells, these were transiently transfected with the respective plasmid construct. FLIM measurements were performed 72 h after transfection. The resulting values for the fluorescence lifetime were displayed on false color scale.

### Supercomplex modeling

For the modeling of respiratory supercomplex CI_1_CIII_2_CIV_1_ we mainly considered well-resolved experimental data, namely EM-5319^8^, and previous models of respiratory supercomplex structures, including all experiments deposited in EMDB (see Supplementary information for details). The best fit was achieved with the following structures: complex I (PDB 4UQ8; EM 2676), cytochrome *bc*_1_ complex dimer (PDB 1PP9) and cytochrome oxidase (PDB 1V55). With electron density maps simulated from X-ray structures with a resolution of 10 Å and average map value 0.1669 the correlation with the experimental density was 0.8346. Fitting and analysis were performed with Chimera v.1.7. For each sequence of labelled COX subunit model structures were generated using structures of GFP (PDB 1GFL) and COX (PDB 1V55) with variable linker conformations. In each case 25 structures were generated with MODELLER v.9.25 and placed in the supercomplex model. All structures of labeled subunits with steric clashes between sEcGFP and membrane or supercomplex proteins were removed (see supplementary information). In each case, several structures were selected to represent available locations for sEcGFP label. Structural visualization and analysis were performed with Pymol v. 1.5.0.3.

### Oxygen consumption measurements

Oxygen consumption of intact HeLa cells was recorded with the Seahorse XF^e^ Extracellular Flux Analyzer (Seahorse Biosciences; North Billerica, MA, USA). ~30.000 cells were seeded into each well of a 96-well Seahorse plate 24 h before the experiment. 60 min before the experiment, cells were washed with XF base medium adjusted to pH 7.4 (Minimal DMEM, 0 mM Glucose, 102353–100 from Seahorse Biosciences), placed in fresh XF base medium pH 7.4 with supplements (1 mM pyruvate, 2 mM L-glutamine, and 5.6 mM D-glucose) and incubated at 37 °C before loading into the XF^e^ Analyzer. Supplements were from Roth. After recording resting respiration in the analyzer, the following chemicals from Seahorse Biosciences were added sequentially to the cells: oligomycin (1 μm), to measure the nonphosphorylating OCR, FCCP (2 μm), to achieve maximal OCR, and antimycin A (0.5 μm) and rotenone (0.5 μm), for determination of the extramitochondrial OCR. For each experiment, three measurements were performed for the resting OCR, three after oligomycin addition, three after FCCP and three after antimycin A plus rotenone with a 2-min interval of recording followed by 2 min of mixing and 2 min of incubation for each measurement.

## Additional Information

**How to cite this article:** Rieger, B. *et al*. Lifetime imaging of GFP at CoxVIIIa reports respiratory supercomplex assembly *in live cells. Sci. Rep.* **7**, 46055; doi: 10.1038/srep46055 (2017).

**Publisher's note:** Springer Nature remains neutral with regard to jurisdictional claims in published maps and institutional affiliations.

## Supplementary Material

Supplementary Information

## Figures and Tables

**Table 1:** Linker length and distances of sEcGFP to the membrane surface.

Construct	AA residues of flexible linker part	Calculated distance [Å] between sEcGFP and IMM surface in a supercomplex	Calculated distance [Å] between sEcGFP and IMM surface in a single complex
CoxVIIc-sEcGFP	7	1–2	5
CoxVIIIa-sEcGFP	11	5	15
CoxVIIIa-Link-sEcGFP	27	50	50
CoxIV-sEcGFP	15	44	44
CII-sEcGFP	7	—	15

Number of amino acid (AA) residues belonging to the flexible linker part between sEcGFP and C-termini of different subunits (see also Supplementary Fig. 4a). Distances [Å] between N-terminus of sEcGFP and the AA residue directly at the inner membrane surface at the different subunits.
