# Supplementary material for: Lifetime imaging of GFP at CoxVIIIa reports respiratory supercomplex assembly in live cells
Source: Sci Rep. 2017 Apr 6;7:46055. doi: 10.1038/srep46055 (PMC5382582; doi:10.1038/srep46055)
Supplement: Supplementary Information [file srep46055-s1.pdf]

Lifetime imaging of GFP at Cox8a reports respiratory supercomplex assembly *in live* cells

Bettina Rieger[1,2], Daria N. Shalaeva[3], Anna-Carina Söhnel[1,2], Wladislaw Kohl[2], Patrick Duwe[1], Armen Y. Mulkidjanian[3,4,5], Karin B. Busch[1,2]

[1]Institute of Molecular Cell Biology, School of Biology, University of Münster, D-48149 Münster, Germany; [2]Mitochondrial Dynamics Group, School of Biology, University of Osnabrueck, D-49076 Osnabrueck, Germany; [3]School of Physics, University of Osnabrueck, D-49069 Osnabrueck, Germany; [4]School of Bioengineering and Bioinformatics, M.V. Lomonosov Moscow State University, 119992, Moscow, Russia, and [5]A.N. Belozersky Institute of Physico-Chemical Biology, M.V. Lomonosov Moscow State University, 119992, Moscow, Russia.

*Corresponding author:

Prof. Dr. Karin Busch

Institute of Molecular Cell Biology

School of Biology

WWU University of Münster

Schloßplatz 5

48149 Münster, Germany

Phone: +49-(0) 151-52738251

Mail: buschkar@.uni-muenster.de

Supplementary Figures

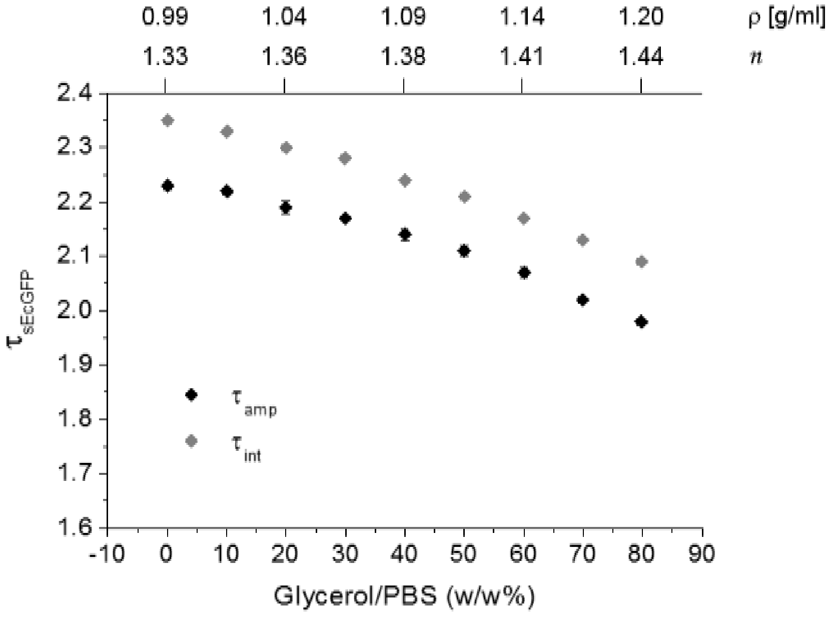

Supplementary Figure S1. Relation of fluorescence lifetime of sEcGFP in aqueous solution with increasing glycerol content to the refractive index *n* and mass density *p*. Determination of τ values of soluble sEcGFP in aqueous solutions with increasing glycerol content at 37°C. τ values (mono-exponential fit) and values for τ_{amp} = ((τ1·A1) + (τ2·A2)) / (A1+A2) and τ_{int} = (($\tau1^2$·A1) + ($\tau2^2$·A2)) / ((τ1·A1) + (τ2·A2)) (bi-exponential fit) are means (± s.d.) of at least 3 independent measurements. Refractive indices (n) of aqueous buffer solutions with increasing glycerol content at 37°C were determined as well and plotted on an additional x axis together with the corresponding mass density in g/ml according to [1]. Note that for most of the data points the error bars are smaller than the symbols.

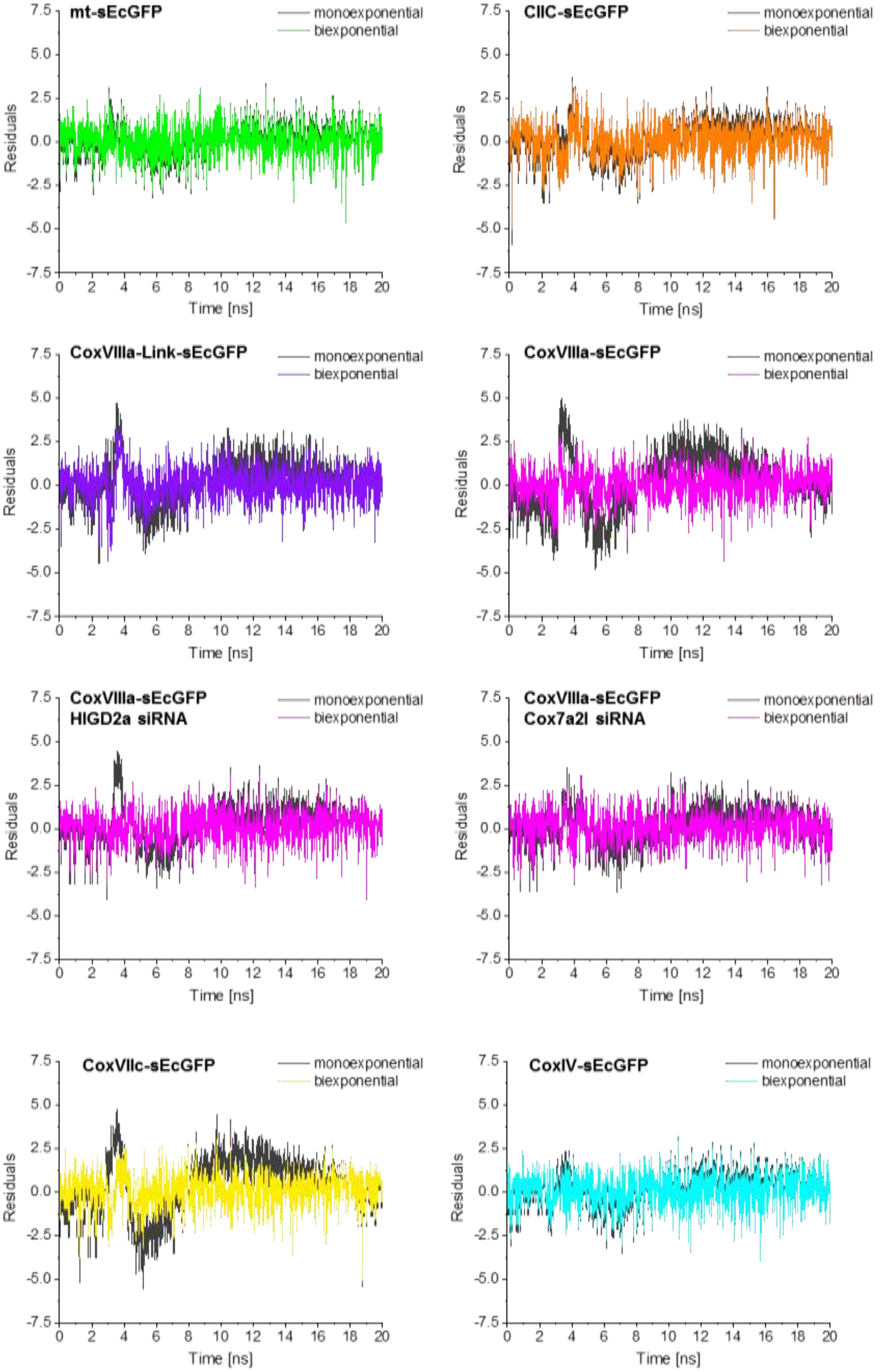

Supplementary Figure S2. Residuals of mono- vs. bi-exponential fitting curves of sEcGFP fluorescence lifetime decay.

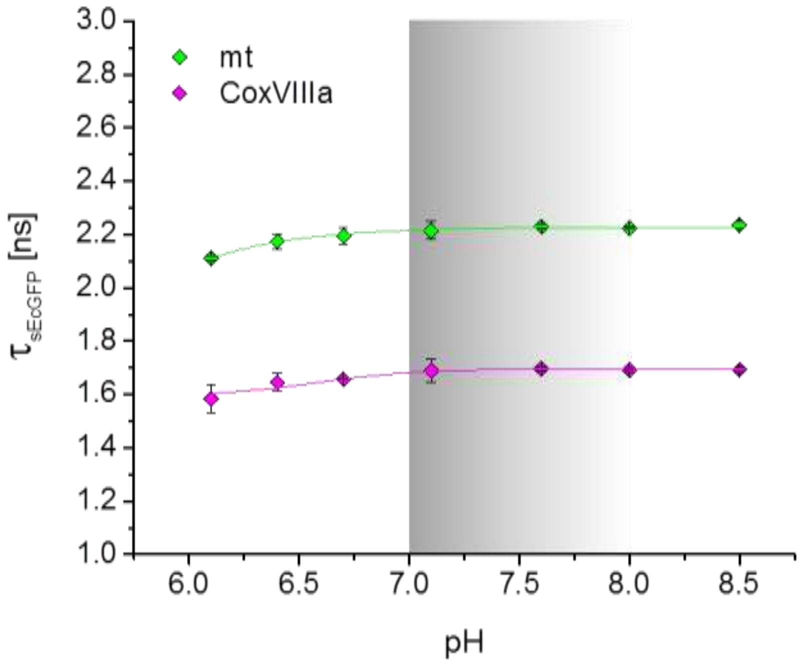

Supplementary Figure S3. Matrix and intermembrane space nano-environments evoke different sEcGFP fluorescence lifetimes. Cells were permeabilized and incubated in buffers (20mM MES, 20mM BES or 20mM HEPPSO) with different pH in the presence of 10 µM CCCP plus 1 µM nigericin. To exclude any interference with mitochondrial activity, oxidative phosphorylation was inhibited by treatment with 5 µg/ml oligomycin. Calibration of fluorescence lifetime for pH (physiological range = IMS pH ca. 7, matrix pH ca. 8) was performed (mt-sEcGFP = green; CoxVIIIa-sEcGFP = pink). Data points represent means, error bars represent s.e.m. (3 biological replicates).

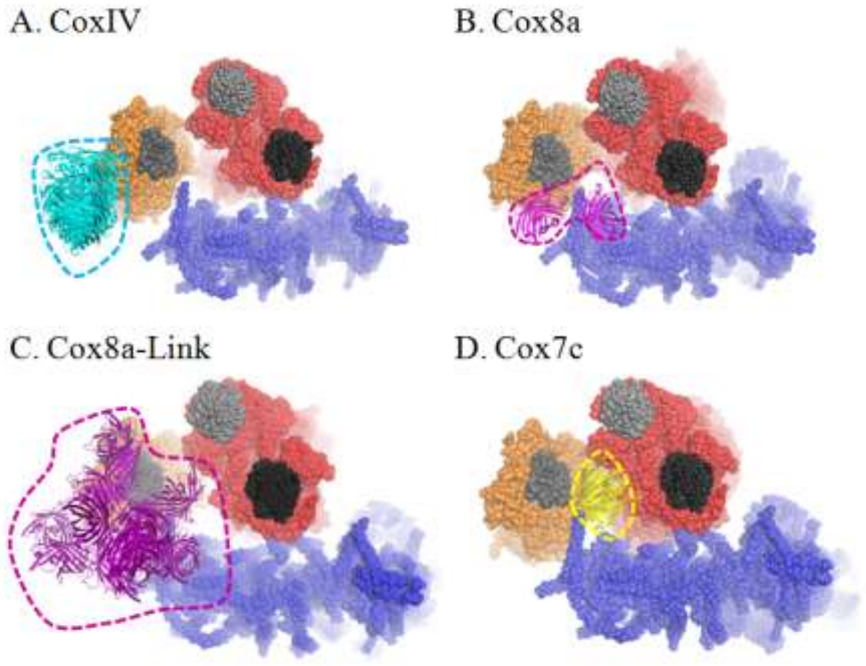

Supplementary Figure S4. Modelling of different sEcGFP conformations in sEcGFP-CIV fusion constructs. Complex I is shown in blue, cytochrome *bc₁* complex dimer is shown in red, cytochrome oxidase is shown in orange, cytochrome *c* molecules shown in the shades of gray. The variety of allowed linker conformations is shown for the fusion constructs and highlighted by dashed lines.

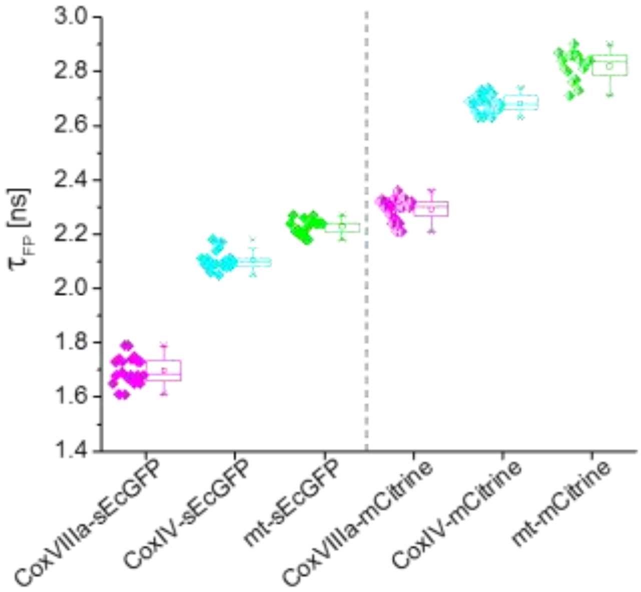

Supplementary Figure S5. Fluorescence lifetime of CoxVIIIa-mCitrine and CoxIV-mCitrine in comparison to fluorescence lifetime of CoxVIIIa-sEcGFP and CoxIV-sEcGFP under same conditions. FP = fluorescent protein; one data point per cell, s.d.; ~20 cells (n = 2 replicates). Statistics: ***, *P*=0.001 compared to control (ANOVA one-way).

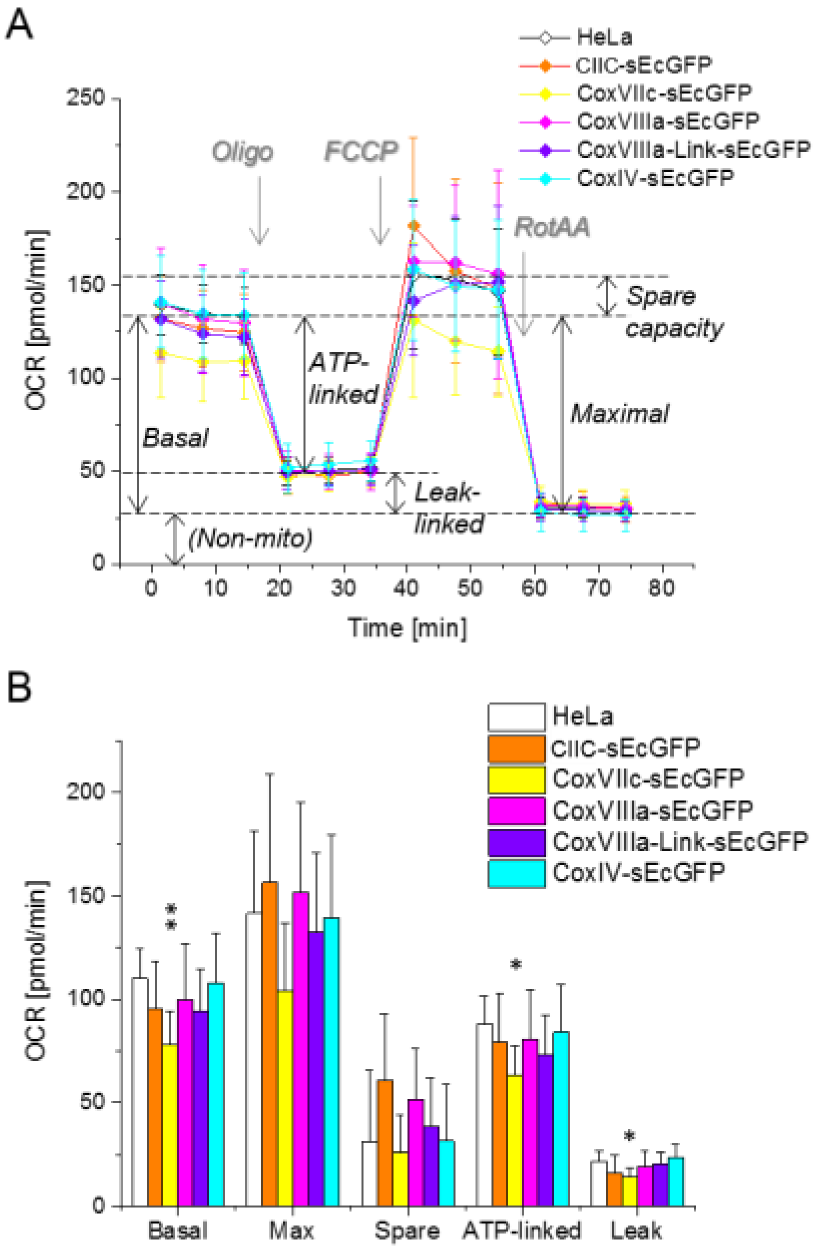

Supplementary Figure S6: Respiratory efficacy of different HeLa cell lines stably expressing sEcGFP fusion constructs. a, oxygen consumption rate (OCR) determined by an Extracellular Flux Analyzer (® Seahorse XF96e). After recording resting respiration, the following chemicals were added sequentially to the cells: oligomycin (Oligo, 1 µM), to measure the non-phosphorylating OCR, FCCP (2 µM) to achieve maximal OCR, and antimycin A (AA, 0.5 µM) and rotenone (Rot, 0.5 µM) for determination of the extra-mitochondrial OCR. Dashed lines and double arrows explain parameters for calculation (representative for untransfected HeLa cells) **b,** Calculation of basal, maximal, ATP-linked and proton leak-linked respiration as well as spare respiratory capacity. Mean values ± s.d. (27 wells per cell line, n = 3 independent experiments). Statistics: no significant differences compared to untransfected HeLa cells for all stable cell lines tested (P≥0.05, ANOVA one-way).

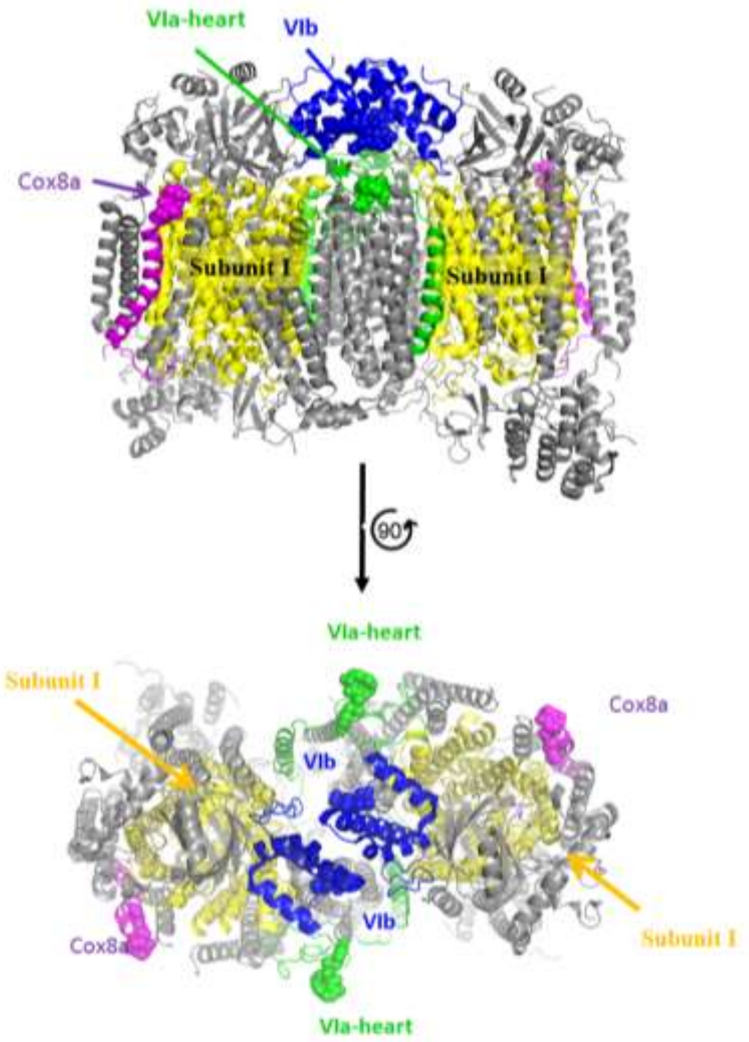

Supplementary Figure S7. Structure of CIV homo-dimer with inward and outward subunits labeled. Top view: subunit I in yellow, CoxVIIIa in pink, subunit CoxVIa in green and subunit CoxVIb in blue (UniProtKB AC: P07471 Blue: Chains H, U, Cytochrome c oxidase polypeptide VIb, UniProtKB AC: P00429). The C-termini of CoxVIIIa subunits in detergent-solubilized, purified dimers are on opposite sides of the homo-dimer, subunits VIa and VIb are probably building the monomer monomer contact side. [2,3]

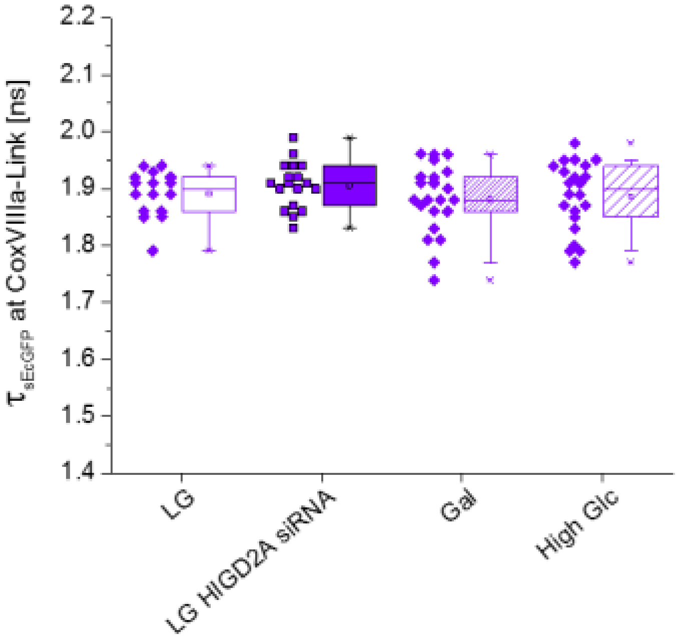

Supplementary Figure S8. Insertion of a longer linker between sEcGFP and CoxVIIIa (CoxVIIIa-Link) results in insensitivity to changes in SC assembly factors.

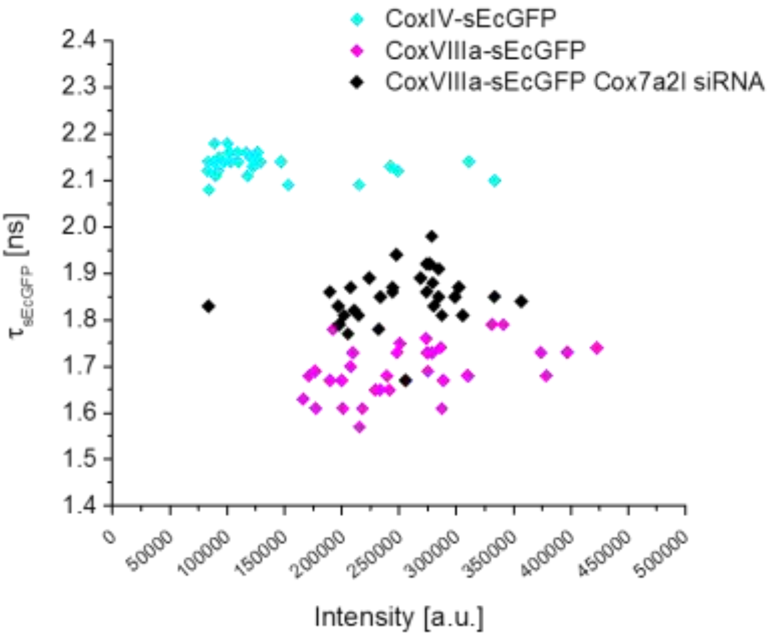

Supplementary Figure S9. Time-resolved measurements are insensitive to the fluorescence intensity respectively the expression level of the construct. Average fluorescence lifetimes related to mean intensities of sEcGFP fused to CoxIV (cyan) or CoxVIIIa (pink). For disruption of supercomplexes, CoxVIIIa-sEcGFP cells were transiently transfected (twice) with siRNA directed against supercomplex assembly factor Cox7a2l (black) as described before. One data point represents the mean τ per cell (~20 cells, 3 independent assays).

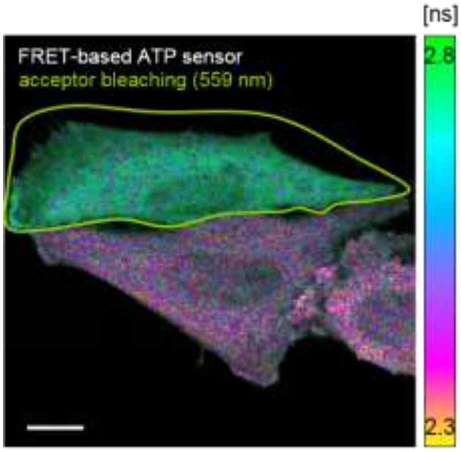

Supplementary Figure S10. FLIM of cells transfected with ATeam. ATeam is a fluorescence resonance energy transfer (FRET)-based indicator for ATP,[4] here with Clover as donor and mRuby as acceptor. Fluorescence lifetime images were recorded including cells with donor only (acceptor bleached) and donor and acceptor. Scale bar: 10 µm.

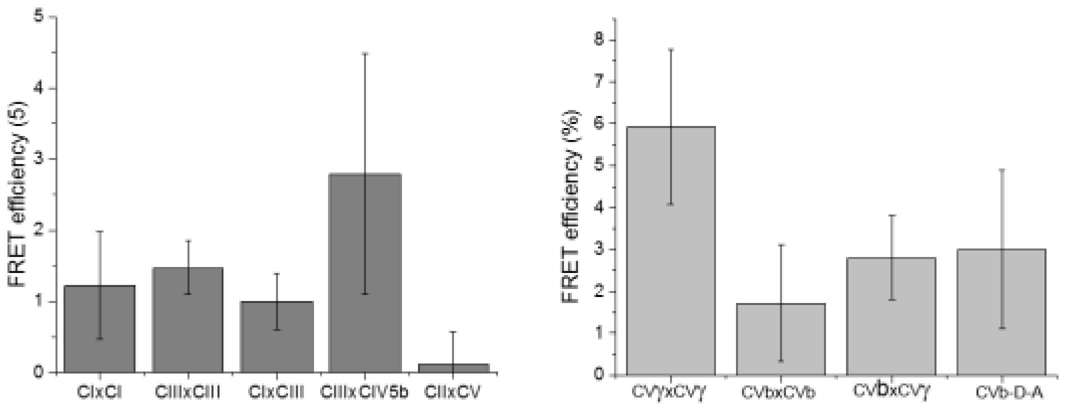

Supplementary Figure S11. FLIM/FRET of cells co-transfected with different OxPhos subunits. a, mEGFP was used as donor and DsRed as acceptor. Complex I (CI) was labeled at subunit 30kD, complex III was labeled at subunit k, complex IV at subunit CoxVb, complex II at subunit B (CII) and ATP synthase at subunit γ (CV). **b**, To check for dimerization of complex V, the following FRET pairs were tested: subunit γ-mEGFP x subunit γ-DsRed (CVγxCVγ, first donor, then acceptor construct), subunit b x subunit b, subunit b x subunit γ. CVb-D-A is a tandem construct of subunit b-eGFP-DsRed where intramolecular FRET can be expected. At least, two independent assays per combination were performed. FRET efficiency was calculated as $E = (1-\tau_{DA}/\tau_D)*100$. Fluorescence lifetime images were recorded in cells 6 days after co-transfection and within one image half, the acceptor was completely bleached to obtain the reference lifetime for the donor in absence of the acceptor.

Supplementary material and methods

***In situ* pH calibration for determination of the pH dependence of fluorescence lifetime.** For pH settings, stable cell lines CoxVIIIa-sEcGFP and mt-sEcGFP were perfused initially for 3–5 min with PBS with $MgCl_2$ and $CaCl_2$ (D8662, pH 7.3). Then, PBS was exchanged by MES (2-(N-morpholino)ethanesulfonic acid, 20mM, pH 5.6-6.1), BES (N,N-bis(2-hydroxyethyl)-2-aminoethanesulfonic acid, 20 mM, pH 6.1-7.6) or HEPPSO (4-(2-hydroxyethyl)-piperazine-1-(2-hydroxy)-propanesulfonic acid, 20 mM, pH 7.1-8.0) buffer prior adjusted to the desired pH by 1 M HCl or NaOH, and supplemented with the following compounds: 125 mM KCl, 20 mM NaCl, 0.5 mM $CaCl_2$, 0.5 mM $MgSO_4$, as well as 10 µM carbonyl cyanide p-chlorophenyl hydrazone, 1 µM nigericin, 5 µg/ml oligomycin. Fluorescence lifetime of sEcGFP was recorded within 15-45 min of incubation in pH adjusted buffers as described in the main methods. Nigericin, CCCP and oligomycin were purchased from Enzo Life. MES was purchased from Biomol, BES and HEPPSO from Sigma. Other commonly used chemicals were purchased from Roth. All measurements were performed at 37°C.

Construction of mCitrine fusion constructs. For eukaryotic expression of the yellow fluorescent protein (YFP) mutant mCitrine (S65G/V68L/Q69M/S72A/T203Y), the full-length protein-coding region of superecliptic pHluorin (F64L/S65T/S147D/N149Q/V163A/S175G/S202F/ Q204T/A206T) (a gift from Jürgen Klingauf) was inserted by PCR amplification into a modified pSEMS-26 m vector from NEB Biosciences. For determination of the fluorescence lifetime in the matrix bulk, the mitochondrial targeting sequence of the mitochondrial processing peptidase (MPP), consisting of the N-terminal 60 amino acid residues (MPP60, referred to as mt), was included behind the CMV promoter and at the 3'end of sEcGFP in this vector resulting in mt-sEcGFP. This targeting sequence is likely removed from the fusion construct by MPP, leaving purely sEcGFP in the matrix. For measurements in the IMS, CoxVIIIa- and CoxIV-mCitrine were assembled behind the CMV promoter and at the 3'end of sEcGFP in the sEcGFP-vector. The respective subunits of OXPHOS complexes were fused with their C-termini to the N-terminus of mCitrine.

Determination of fluorescence intensity. The fluorescence intensity was recorded using the confocal laser scanning microscope (FluoView FV1000, Olympus) with 60x oil-immersion objective (UPLSAPO oil, NA 1.35, N/0.17/FN26.5, Olympus) and a continuous 488 nm laser. Imaging was performed at 37°C 5% CO_2. The temperature at the objective was hold by an objective heater (BIOPTECHS). Determination of mean fluorescence intensity (all mitochondria per cell) was performed using ImageJ software (NIH Image, http://rsb.info.nih.gov/nih image/index.html). For thresholding, the otsu filter was used as a mask for mitochondria and the background was set to NaN.

Oxygen consumption measurements. Oxygen consumption of intact HeLa cells was recorded with the Seahorse XF^{e}96 Extracellular Flux Analyzer (Seahorse Biosciences; North Billerica, MA, USA). 30.000 cells were seeded into each well of a 96-well XF cell culture microplate 24 h before the

experiment. 60 min before the experiment, cells were washed with XF assay medium made of XF base medium (Minimal DMEM, 0 mM Glucose, 102353-100 from Seahorse Biosciences) with supplements (1 mM pyruvate, 2 mM L-glutamine, and 5.6 mM D-glucose – adjusted to pH 7.4), placed in fresh XF assay medium and incubated at 37°C before loading into the XF^e Analyzer. Supplements were from Roth. After recording resting respiration in the analyzer, the following chemicals from Seahorse Biosciences were added sequentially to the cells: oligomycin (1 µM), to measure the non-phosphorylating OCR, FCCP (2 µM), to achieve maximal OCR, and antimycin A (0.5 µM) and rotenone (0.5 µM), for determination of the extramitochondrial OCR. Three measurements (cycles) were performed for the resting OCR, three after oligomycin addition, three after FCCP and three after antimycin A plus rotenone with a 3-min interval of recording and a 3-min interval of mixing for each measurement.

SC modeling. For the modeling of the supercomplex we used Cryo-EM density map EMDB 5319[5]. For particular proteins, we used crystal structures: cytochrome bc_1 complex PDB 1PP9, 2.1Å,[6] cytochrome oxidase PDB 1V55, 1.9Å. For the Complex I we had compared all available to date structures and superposed them with each other. From this comparison, we chose 3 most diverse structures with high resolution for further consideration: PDB ID 4UQ8, 4WZ7 and 5LDW. Finally, we compared our final model with other cryo-EM maps and corresponding atomic models. The best fit to original Cryo-EM density map EMDB 5319[5] was achieved with Complex I structure PDB 4UQ8 (EM 2676), 4.95Å.[7] For visualization and manipulation with cryo-EM data and large atomic protein structures we used the UCSF Chimera v.1.7 software.[8]

Supplementary Table 1. All available in EMDB density maps of mammalian respirasome

EMD ID	Res., Å	PDB ID	Composition	Organism	Reference
8130	5.8	5J4Z	I+III2+IV	*Ovis aries*	[9]
8128	6.7	5J7Y	I+III2+IV	*Ovis aries*	[9]
8129	7.8	5J8K	I+III2	*Ovis aries*	[9]
4108	10.4	N/A	I+III2+IV	*Bos taurus*	[10]
4109	9.9	N/A	I+III2	*Bos taurus*	[10]
1876	19	2YBB	I+III2+IV	*Bos taurus*	[11]
5319	22	N/A	I+III2+IV	*Bos taurus*	[5]
1318	32	N/A	I+III2+IV	*Bos taurus*	[12]
*9534	5.4	*5GPN	I+III2+IV	*Sus scrofa*	[13]

* To the date (November 2016) the structures described in the paper[13] are not available in PDB and EMDB databases.

Supplementary Table 2. Fit quality estimates for different full-atom respirasome models.

**PDB**	**4uq8-1pp9-1v55**		**4hea-1pp9-1v55**		**5ldw-1pp9-1v55**	
EMD	Correlation	Avg. map vol.	Correlation	Avg. map vol.	Correlation	Avg. map vol.
8130	0.7536	0.1014	0.6578	0.07163	0.728	0.09174
4108	0.8119	0.0794	0.8035	0.07109	0.8141	0.0778
5319	**0.8315**	**0.1627**	0.8357	0.1546	0.8324	0.1603

**PDB**	**5j4z**		**2ybb**	
EMD	Correlation	Avg. map vol.	Correlation	Avg. map vol.
8130	0.9107*	0.1565*	0.6366	0.06654
4108	0.7695	0.079	0.7982	0.07139
5319	0.7785	0.157	0.8211	0.1547

The correlation of fit ranges from –1 to 1, and was calculated using simulated map with 5Å resolution for the models. Average map volume calculated from density map values averaged over the center positions of all the fit atoms, thus a higher value means the atoms sit in higher density, indicating a better fit. * Density map and full-atom model from the same work.[9]

Overall, our model shows high similarity to available experimental models, especially one of the newest models with the highest available to date resolution: model of the “tight” respirasome PDB 5J4Z with resolution 5.8 Å.[9]

We used "Orientation of Proteins in Membranes" (**OPM**) database[14] to obtain protein structures with assigned membrane limits (available at http://opm.phar.umich.edu). We used this data to ensure correct orientation of each protein in membrane with respect to other proteins in the supercomplex model. The OPM service provides spatial arrangements of membrane proteins with respect to the hydrocarbon core of the lipid bilayer, but for visualization we marked borders between the membrane lipids and the solution.

Additionally, we have placed a cytochrome *c* molecule structures in each of the three known binding sites in the supercomplex: one on the cytochrome oxidase monomer, and two on the cytochrome bc_1 complex dimer. The X-ray structure of the cytochrome bc_1 complex dimer (PDB 1PP9)[6] contains only one cytochrome *c* molecule, the second one was places symmetrically. On the surface of the

cytochrome oxidase the cytochrome *c* molecule was placed manually in agreement with known particular residue interactions. [15-17]

For each sequence of labeled COX subunit model structures were generated using structures of GFP (PDB 1GFL)[18] and COX (PDB 1V55)[19] with variable linker conformations. In each case 25 structures were generated with MODELLER v.9.25 and placed in the supercomplex model. For modeling the linker between two proteins each model was first optimized with the variable target function method with conjugate gradients, and then refined using molecular dynamics (a MODELLER build-in extension) with simulated annealing. All structures of labeled subunits with steric clashes between sEcGFP and membrane or supercomplex proteins were removed. In each case a single structure was selected to represent the position of the sEcGFP label. Structure visualization and analysis were performed with Pymol v. 1.5.0.3.[20] All proteins are shown as solvent-accessible surface with solvent radius 3Å. To create models of supercomplex with sEcGFP label attached by a protein linker to particular subunits we used homology modeling methods implemented in MODELLER v9.[21] First, we created alignments of a labeled subunit sequences with sequences matching known structures: corresponding native subunit of cytochrome oxidase and the GFP. We used Clustal Omega[22] to create all alignments.

To create structures of sEcGFP-labeled subunits of supercomplex proteins we took the structure of the GFP and the sequence of the sEcGFP with protein linker and only few first residues of respective supercomplex protein subunit. With this set-up we created 25 models with variable conformations of the linker. Then the sEcGFP-linker structures were attached to the corresponding end of the protein chain of each model. The loop optimization by MODELLER allowed us to create a wide range of geometrically reasonable conformations of the linker. These models were superposed with corresponding subunits in the supercomplex; all models that created steric clashes between sEcGFP and the supercomplex proteins or the membrane were removed (see supplementary Figure XXX). The fraction of models, fitting into the supercomplex structure without clashing with other proteins or the membrane corresponds to the conformational space, available for the linker in a particular location (Table YYY). This number is bigger for mobile labels, which have longer linkers, and are exposed to the solution; and smaller for labels, attached by a short linker and/or surrounded by other proteins, and thus have restricted mobility. To give quantitative estimates of relative mobility of each linker, we also considered the number of “free” residues in each fusion construct, as a number of residues, mainly responsible for the linker and label mobility. Thus, we counted all residues between original subunit and the fluorescent label, not participating in any secondary structure and not restricted by a hydrogen bond or a salt bridge. Finally, we estimated the maximum distance between the sEcGFP label and nearest surface (of membrane or supercomplex protein) achieved with a fully stretched conformation of the linker (Supplementary Table 3)

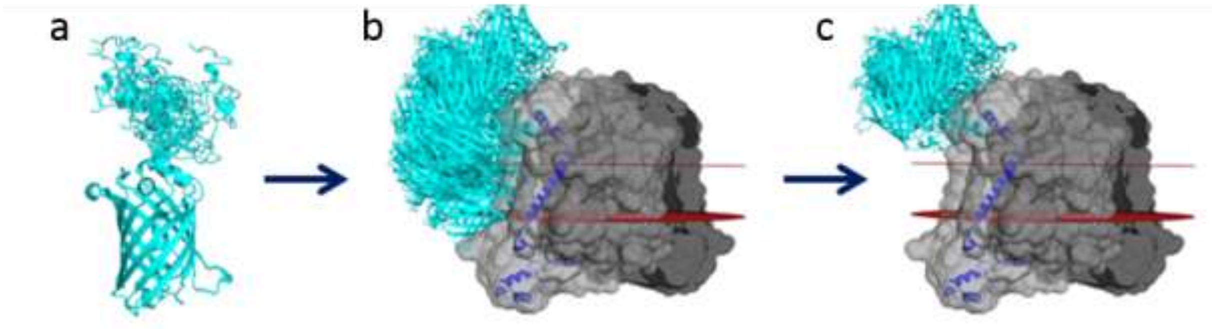

Supplementary Figure S12. Stages of modeling structure of sEcGFP fused with subunits of COX and CIII, shown for subunit IV of COX. a, Using the structure of GFP as a template, 25 models with different conformations of linker are created with MODELLER using build-in molecular dynamics to generate reasonable conformations of the linker. **b**, All generated models are attached to the structure of the whole protein (here, COX dimer) by superposition of the first 3 residues of the linker with corresponding 3 C-terminal residues of the labeled subunit. **c**, All model structures of the fused subunit are inspected manually and structures, overlapping with membrane or protein are removed. Thus, only models with reasonable conformations of the linker and realistic possible placement of the sEcGFP are left. COX monomers are shown in different shades of gray, membrane location is indicated by red dots, labeled subunit IV of COX is shown as dark blue cartoon, models of sEcGFP with a linker are shown in cyan cartoon.

Supplementary Table 3. Mobility of sEcGFP fused with different subunits of cytochrome oxidase. Comments in the text.

Subunit	Max. distance	Number of models	Max. distance	Number of models	Linker length
	In COX dimer		In Supercomplex		
CoxIV	44 Å	14	44 Å	14	15
Cox8a	15 Å	6	5 Å	2	11
Cox8a-Link	50 Å	14	50 Å	12	27
Cox7c	5 Å	7	2 Å	2	7

Supplementary discussion

Fluorescence lifetimes of sEcGFP decreases with increasing glycerol content of aqueous solutions

To show that the radiation lifetime is a function of the inverse refractive index in square as predicted by Strickler-Berg[23], the average fluorescence lifetime (amplitude weighted lifetime τ_{amp}) of sEcGFP was determined in increasing glycerol concentrations. However, measurements in mixtures of more than 90% glycerol were not realizable in an accurate way since homogeneous solubilisation was not possible any more. Nevertheless, it becomes clear that the fluorescence lifetime of purified sEcGFP in 90% - 100% glycerol/PBS (w/w) would not be decreased to the lifetime of CoxVIIIa-sEcGFP (τ~1.7 ns) nor could the refractive index be reduced to the value of proteins (n~1.6,).[24] As scattering in the solution, which is more dramatic for the amplitude than the intensity weighted lifetime, might reduce the lifetime, we analyzed τ_{int} as well and compared it to the τ_{amp} values. We observed that

curve progression of these lifetimes was analogical. Thus, determination of τ_{amp} in solution was reliable.

Supplementary references

1 Association, G. P. *Physical Properties of Glycerine and Its Solutions*, 1963).

2 Musatov, A. & Robinson, N. C. Cholate-induced dimerization of detergent- or phospholipid-solubilized bovine cytochrome C oxidase. *Biochemistry* **41**, 4371-4376 (2002).

3 Sedlak, E. & Robinson, N. C. Phospholipase A(2) digestion of cardiolipin bound to bovine cytochrome c oxidase alters both activity and quaternary structure. *Biochemistry* **38**, 14966-14972 (1999).

4 Imamura, H. *et al.* Visualization of ATP levels inside single living cells with fluorescence resonance energy transfer-based genetically encoded indicators. *Proceedings of the National Academy of Sciences of the United States of America* **106**, 15651-15656,(2009).

5 Dudkina, N. V., Kudryashev, M., Stahlberg, H. & Boekema, E. J. Interaction of complexes I, III, and IV within the bovine respirasome by single particle cryoelectron tomography. *Proceedings of the National Academy of Sciences* **108**, 15196-15200, doi:10.1073/pnas.1107819108 (2011).

6 Huang, L. S., Cobessi, D., Tung, E. Y. & Berry, E. A. Binding of the respiratory chain inhibitor antimycin to the mitochondrial bc(1) complex: A new crystal structure reveals an altered intramolecular hydrogen-bonding pattern. *J Mol Biol* **351**, 573-597, (2005).

7 Vinothkumar, K. R., Zhu, J. P. & Hirst, J. Architecture of mammalian respiratory complex I. *Nature* **515**, 80-+, d (2014).

8 Pettersen, E. F. *et al.* UCSF Chimera--a visualization system for exploratory research and analysis. *Journal of Computational Chemistry* **25**, 1605-1612, (2004).

9 Letts, J. A., Fiedorczuk, K. & Sazanov, L. A. The architecture of respiratory supercomplexes. *Nature* **537**, 644-648, (2016).

10 Sousa, J. S., Mills, D. J., Vonck, J. & Kuhlbrandt, W. Functional asymmetry and electron flow in the bovine respirasome. *Elife* **5**, (2016).

11 Althoff, T., Mills, D. J., Popot, J. L. & Kuhlbrandt, W. Arrangement of electron transport chain components in bovine mitochondrial supercomplex I1III2IV1. *EMBO J* **30**, 4652-4664, (2011).

12 Schafer, E., Dencher, N. A., Vonck, J. & Parcej, D. N. Three-dimensional structure of the respiratory chain supercomplex I1III2IV1 from bovine heart mitochondria. *Biochemistry* **46**, 12579-12585, doi:10.1021/bi700983h (2007).

13 Gu, J. *et al.* The architecture of the mammalian respirasome. *Nature* **537**, 639-643, doi:10.1038/nature19359 (2016).

14 Lomize, M. A., Lomize, A. L., Pogozheva, I. D. & Mosberg, H. I. OPM: orientations of proteins in membranes database. *Bioinformatics* **22**, 623-625, (2006).

15 Roberts, V. A. & Pique, M. E. Definition of the interaction domain for cytochrome c on cytochrome c oxidase - III. Prediction of the docked complex by a complete, systematic search. *J Biol Chem* **274**, 38051-38060, (1999).

16 Wang, K. F. *et al.* Definition of the interaction domain for cytochrome c on cytochrome c oxidase - II. Rapid kinetic analysis of electron transfer from cytochrome c to Rhodobacter sphaeroides cytochrome oxidase surface mutants. *J Biol Chem* **274**, 38042-38050, (1999).

17 Zhen, Y. J., Hoganson, C. W., Babcock, G. T. & Ferguson-Miller, S. Definition of the interaction domain for cytochrome *c* on cytochrome c oxidase - I. Biochemical, spectral, and kinetic characterization of surface mutants in subunit II of *Rhodobacter sphaeroides* cytochrome aa_3. *J Biol Chem* **274**, 38032-38041, doi:DOI 10.1074/jbc.274.53.38032 (1999).

18 Yang, F., Moss, L. G. & Phillips, G. N., Jr. The molecular structure of green fluorescent protein. *Nat Biotechnol* **14**, 1246-1251 (1996).

19 Tsukihara, T. *et al.* The low-spin heme of cytochrome c oxidase as the driving element of the proton-pumping process. *P Natl Acad Sci USA* **100**, 15304-15309, doi:10.1073/pnas.2635097100 (2003).
20 Schrodinger, LLC. *The PyMOL Molecular Graphics System, Version 1.3r1* (2010).
21 Sali, A., Potterton, L., Yuan, F., van Vlijmen, H. & Karplus, M. Evaluation of comparative protein modeling by MODELLER. *Proteins* **23**, 318-326, (1995).
22 Sievers, F. *et al.* Fast, scalable generation of high-quality protein multiple sequence alignments using Clustal Omega. *Mol Syst Biol* **7**, 539, (2011).
23 Strickler, S. J. & Berg, R. A. Relationship between Absorption Intensity and Fluorescence Lifetime of Molecules. *Journal of Chemical Physics* **37**, 814-&, doi:Doi 10.1063/1.1733166 (1962).
24 McMeekin, T. L., Groves, M. L. & Hipp, N. J. in *Amino Acids and Serum Proteins Advances in Chemistry* (ed J. Stekol) Ch. 4, 54-66 (American Chemical Society, 1964).
